# Mobile Health Technology for Enhancing the COVID-19 Response in Africa: A Potential Game Changer?

**DOI:** 10.4269/ajtmh.20-0506

**Published:** 2020-05-29

**Authors:** Jean B. Nachega, Rory Leisegang, Oscar Kallay, Edward J. Mills, Alimuddin Zumla, Richard T. Lester

**Affiliations:** 1 Department of Medicine and Centre for Infectious Diseases, Faculty of Medicine and Health Sciences, Stellenbosch University, Cape Town, South Africa;; 2 Departments of Epidemiology and International Health, Johns Hopkins Bloomberg School of Public Health, Baltimore, Maryland;; 3 Departments of Epidemiology, Infectious Diseases and Microbiology, Center for Global Health, University of Pittsburgh Graduate School of Public Health, Pittsburgh, Pennsylvania;; 4 Family Clinical Research Unit (FAMCRU), Faculty of Medicine and Health Sciences, Stellenbosch University, Cape Town, South Africa;; 5 Division of Pharmacometrics, Department of Pharmaceutical Biosciences, Uppsala University, Uppsala, Sweden;; 6 School of Public Health, University of Rwanda, Kigali, Rwanda;; 7 Erasme Hospital, Université Libre de Bruxelles, Brussels, Belgium;; 8 Division of Infection and Immunity, Centre for Clinical Microbiology, University College London, London, United Kingdom;; 9 National Institute for Health Research Biomedical Research Centre, University College London Hospitals, London, United Kingdom;; 10 Division of Infectious Diseases, Department of Medicine, University of British of Columbia, Vancouver, Canada

The WHO Africa Region is experiencing an increase in the number of novel COVID-19 cases. As of May 20, 2020, 63,521 cases with 1,796 deaths (2.8% case fatality) have been reported from 45 countries.^1^ Although these numbers are small compared with those in United States or Europe, the WHO recently estimated that up to 190,000 people could die of COVID-19 in Africa if the pandemic is not controlled.^2^ These projections are threatening the already overstretched health services in Africa, where governments have been implementing mitigating strategies to flatten epidemic curves at manageable levels. These include education, personal hygiene practices, social distancing, travel bans, and partial or total lockdowns.^3^ However, as lockdowns and social distancing measures are currently being lifted in stages by most African countries, governments will need to ensure that public health infrastructure and needed resources are put in place for community surveillance to identify cases and clusters of new infections through active case finding, large-scale testing, and contact tracing.

Cost-efficient testing strategies with rapid turnaround and community-based contact-tracing approaches are cornerstones for containment during epidemics. To do so at scale and over the anticipated prolonged course of this pandemic, African countries will need to capitalize on digital health innovations.^4–6^ The Global System for Mobile Communication Association reports that 50% of Africans own mobile phones and that 39% are internet-connected, numbers which are rapidly increasing, and approach 80% access when phone-sharing is considered.^7^ Mobile phone technology (mHealth) platforms are effective in improving service delivery and outcomes for many health conditions in Africa and globally, including HIV infection, tuberculosis, and chronic noncommunicable diseases.^4–7^

In the context of COVID-19, mHealth solutions offer opportunities to directly support public education, case management, and contact tracing, and to perhaps even provide geolocation and exposure notification.^7,8^ With the support of global mobile technology companies and small and medium enterprises within Africa, mHealth offers opportunities ranging from text messaging to mobile apps to mitigate the spread of COVID-19. The use of mobile phones reduces the need for physical contact, exchange of materials, and movement by health workers, and thus maximizes safety.

**Figure 1.**
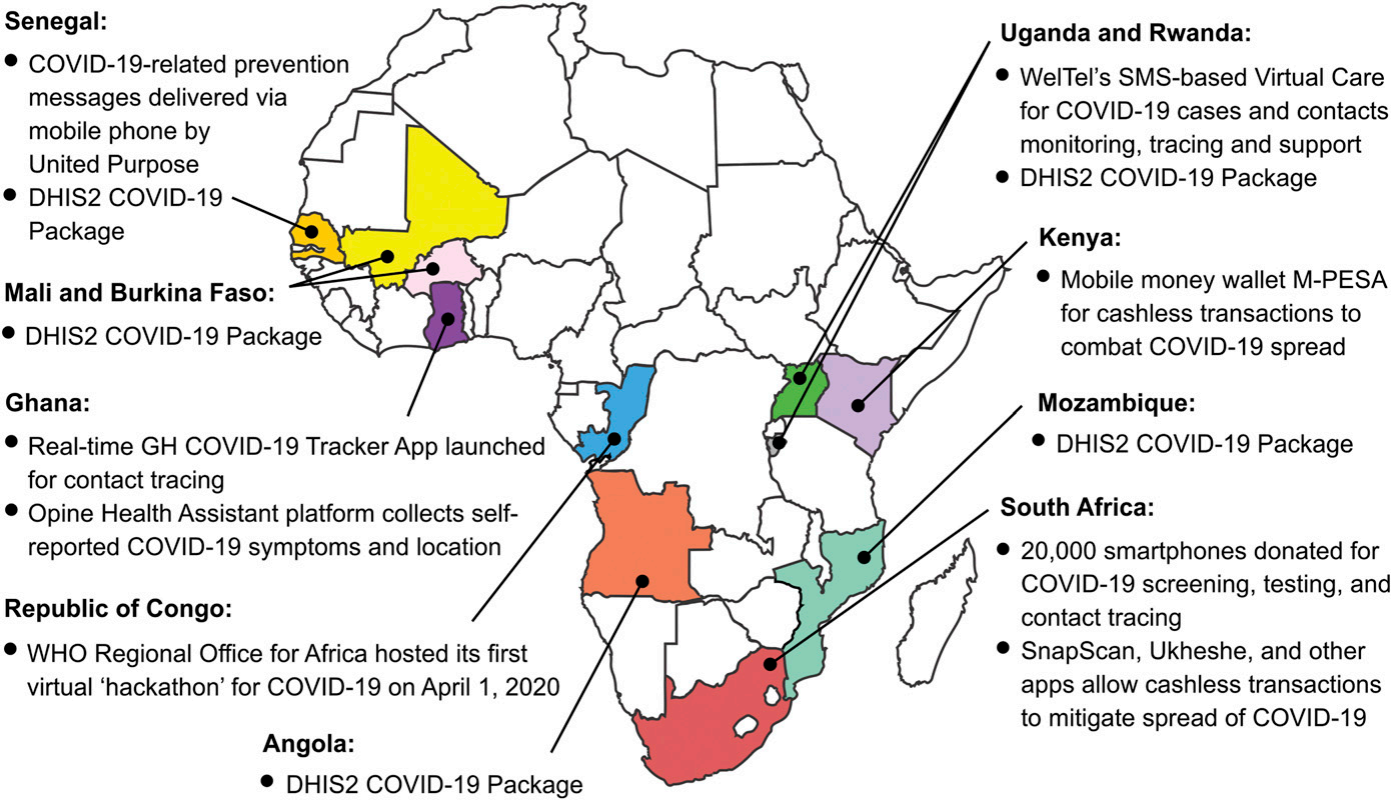
Illustrative COVID-19 mHealth initiatives across Africa (not exhaustive). DHIS2 = District Health Information Software 2.

Several ongoing digital and mobile initiatives related to COVID-19 have been identified across Africa (Figure 1). District Health Information Software 2 is an open-source, web-based health management information system platform already used by 67 low- and middle-income countries. District Health Information Software 2 has a COVID-19–specific application package that several African countries are using for field data collection.^9^ In Rwanda and Uganda, the WelTel virtual care system serves as a real-time remote monitoring platform. COVID-19 cases and contacts in home isolation receive semi-automated daily text message check-ins via SMS for 2 weeks using an open language format, allowing self-reporting of new symptoms or issues. Responses are viewed by health officials on a dashboard, and patients are triaged much faster than would be the case with traditional field outreach or telephone calls, saving critical human resource capacity. Novel natural language processing computing tools promise to reveal insights into the issues that patients face during home quarantine. The provision of monitoring packaged with interactive support helps people undertake home isolation/quarantine most effectively.^10^ In Ghana, a short USSD code (*920*222#) dialed on mobile phones allows residents to respond electronically to questions about their symptoms, who they have been in contact with, and their travel history. The Opine Health Assistant compiles the results into maps and graphs to make it easier to understand, monitor, and share.^11^ In Senegal, SMS services are used to broadcast good hygiene practices to rural communities to disrupt the spread of COVID-19.^12^ In South Africa, community screening, referral for testing, and communication of results of using an mHealth platform are being rapidly expanded to more than 28,000 trained community health workers.^13^

Mobile phones and apps also support livelihoods and enable remote access to critical services such as education and food. In Kenya, transaction fees for using M-PESA, a cashless, mobile money platform with 20 million users, have been waived to provide a safe method by which to transfer funds within community settings. In South Africa, mobile data costs of accessing some teaching and learning websites have been waived by major cellphone providers to ensure that primary and secondary school and university students can continue to access learning materials. Globally, mobile counseling, support hotline, and social media platforms are assisting with public health information as well as mental health counseling, food relief, domestic violence concerns, and other support. Government and private alignment within these platforms should be encouraged, as oversight by public health agencies will ensure accurate content.

In conclusion, there appears to be a limited window of opportunity in which to contain the spread of COVID-19 in Africa and keep economies afloat. There is a significant body of innovation and evidence to inform mHealth best practices that have emerged from Africa over the past decade.^14–16^ mHealth may be a game changer if it is introduced swiftly and widely in this pandemic. To succeed, barriers to access to and use of mobile phones and the latest technologies need to be defined, and there must be cooperation among all stakeholders to enable rapid deployment and scale-up of promising or evidence-based solutions. If mHealth is rigorously implemented, scaled-up, and evaluated through implementation science, then Africa will reap the benefits of this technology for the remainder of the COVID-19 crisis and be better positioned for future pandemics and for improving all aspects of public health.
